# Clarifying data interpretation and therapeutic classification in ADC clinical trials for lung cancer

**DOI:** 10.1097/JS9.0000000000002676

**Published:** 2025-06-10

**Authors:** Man Sun, Dan Zang, Jun Chen

**Affiliations:** Department of Oncology, The Second Hospital of Dalian Medical University, 467 Zhongshan Road, Shahekou District, Dalian City, Liaoning Province, China

*Dear Editor,*

We read with great interest the article published in International Journal of Surgery by Yijiang He and colleagues[1]. This study analyzed the clinical trial landscape of antibody–drug conjugates (ADCs) in lung cancer by aggregating public datasets, revealing their predominant use in advanced-stage disease and later-line therapies, with growing focus on target-specific precision approaches. As ADCs continue to expand rapidly in this field, the need for standardized clinical classification becomes increasingly critical to ensure appropriate interpretation and application. In this letter, we offer several comments and clarifications regarding the data representation and clinical classification framework proposed in the original article[2].

First, we note that the temporal labeling in Figure B – “from before 2014 to 2024” – is linguistically ambiguous and may obscure whether “before 2014” denotes a specific year, a time range, or an aggregated category. For greater clarity, particularly for international readers, we recommend rephrasing as: “Figure B shows the annual distribution of ADC clinical trials from 2014 to 2024, with all trials initiated before 2014 grouped under ‘pre-2014ʹ.” This revision would improve interpretability and enhance the clarity of data presentation.

Second, in Figure 1D, titled “Main ADC drugs under investigation for lung cancer,” pembrolizumab is incorrectly included. As a PD-1 immune checkpoint inhibitor, pembrolizumab is not an ADC. Although frequently used in combination with ADCs in clinical settings, classifying it as an ADC is mechanistically inaccurate and may mislead readers unfamiliar with their distinct modes of action. Unlike ADCs – comprising a monoclonal antibody covalently linked to a cytotoxic payload – checkpoint inhibitors exert effects via immune modulation without a conjugated cytotoxic component[3]. Additionally, disitamab vedotin and patritumab deruxtecan are listed twice, which may compromise the clarity and accuracy of the data presentation.

Of note, Figure 1E, labeled “Cancer staging distribution,” shows unexpectedly high numbers of early-stage lung cancer cases (Stage I: *n* = 341; Stage II: *n* = 316), which appears inconsistent with the known clinical application of ADCs. In current practice and ongoing trials, ADCs are predominantly studied in Stage III–IV non-small cell lung cancer (NSCLC), particularly in patients with unresectable, advanced, or metastatic disease. To substantiate this concern, we conducted a review of ADC-related lung cancer trials from 2001 to 2025. As shown in Figure 1, among the pooled cases, early-stage patients (Stage I: *n* = 4; Stage II: *n* = 18) were exceedingly rare, while most participants were Stage III (*n* = 399) or Stage IV (*n* = 419). These findings suggest that Figure 1E may have included trials not primarily investigating ADCs or misclassified patient populations, potentially misrepresenting the actual clinical positioning of these agents. We recommend clarifying the inclusion criteria and staging classification to ensure accurate reflection of real-world and trial-based ADC use.

**Figure 1.**
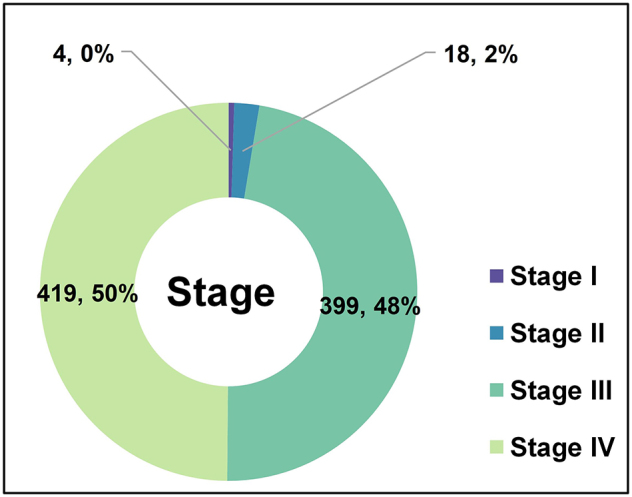
Clinical Stage Distribution of Patients Enrolled in ADC Trials for Lung Cancer (2001–2025).

Donut chart illustrating the distribution of clinical stages among patients enrolled in ADC clinical trials for lung cancer from 2001 to 2025. The majority of participants were at advanced stages, with 399 patients (48%) at Stage III and 419 patients (50%) at Stage IV. In contrast, early-stage enrollment was rare, with only 18 patients (2%) at Stage II and 4 patients (0%) at Stage I. These findings emphasize the predominant use of ADCs in advanced, unresectable, or metastatic lung cancer.Note: This figure was created independently by the letter authors and is not part of the original publication.

In addition, we would like to raise a conceptual concern regarding the classification of therapeutic stages in the manuscript by He *et al*. Figure E appears to conflate neoadjuvant and adjuvant therapies with systemic lines of therapy (LOT), which may lead readers to misinterpret perioperative interventions as part of the treatment continuum for advanced or metastatic disease. According to standard oncologic practice and established guideline definitions (e.g., NCCN, ESMO), LOT refers specifically to systemic therapies administered in the context of unresectable, relapsed, or metastatic disease[4]. Neoadjuvant and adjuvant therapies, while critical for early-stage lung cancer, are not typically counted toward LOT unless recurrence or progression requires systemic re-treatment.

Importantly, we emphasize that the use of ADCs remains investigational, with limited efficacy data and unresolved safety concerns in early-stage lung cancer. Severe adverse events – such as interstitial lung disease, cardiotoxicity, and immune-mediated toxicities – necessitate rigorous evaluation before ADCs can be considered appropriate for neoadjuvant or adjuvant use[5]. In addition, identifying patient subgroups that may truly benefit from perioperative ADC administration remains an open question, given the current lack of predictive biomarkers or validated selection strategies. Therefore, we strongly recommend that perioperative therapies be clearly distinguished from systemic lines of therapy in both the figure and text to avoid overestimating the extent of clinical integration. Given the current balance of risk and benefit, it may not yet be the optimal time to incorporate ADCs into perioperative treatment strategies.

It is commendable that the authors present a comprehensive overview of ADC clinical trials in lung cancer – an evolving therapeutic landscape that warrants continued multidisciplinary effort. This correspondence clarifies key misclassifications in ADC trials and underscores the need for precise therapeutic staging to improve interpretation, ensure cautious clinical integration, and support more accurate trial reporting.

## Data Availability

Not applicable.

## References

[1] He Y Huang W Hong H Li Y Shen Y Qu Y. Clinical trial landscape of lung cancer treatment with ADCs: current perspectives and future directions. Int J Surg Published online May 16, 2025 doi:10.1097/JS9.0000000000002501 40387697

[2] Agha RA Mathew G Rashid R. Transparency in the reporting of artificial intelligence – the TITAN guideline. Prem J Sci Published online 2025. doi:10.70389/PJS.100082.

[3] Wang R Hu B Pan Z. Antibody–drug conjugates (ADCs): current and future biopharmaceuticals. J Hematol Oncol 2025;18:51. 40307936 10.1186/s13045-025-01704-3 PMC12044742

[4] Riely GJ Wood DE Ettinger DS. Non-small cell lung cancer, version 4.2024, NCCN clinical practice guidelines in oncology. J Natl Compr Cancer Netw JNCCN 2024;22:249–74. 38754467 10.6004/jnccn.2204.0023

[5] Heist RS Sands J Bardia A. Clinical management, monitoring, and prophylaxis of adverse events of special interest associated with datopotamab deruxtecan. Cancer Treat Rev 2024;125:102720. 38502995 10.1016/j.ctrv.2024.102720

